# Similar growth potentials of cyanobacteria and algae explain their coexistence in desert soils

**DOI:** 10.1038/s41598-025-30489-1

**Published:** 2025-12-01

**Authors:** Khin Maw Kyi, Elad Levintal, Nina A Kamennaya

**Affiliations:** 1 Kreitman School of Advanced Graduate Studies, Ben-Gurion University of the Negev, Beer Sheva, Israel; 2 Zuckerberg Institute for Water Research, Blaustein Institutes for Desert Research, Ben-Gurion University of the Negev, Sede Boqer Campus, Midreshet Ben-Gurion, Israel; 3 Goldman Sonnenfeldt School of Sustainability and Climate Change, Ben-Gurion University of the Negev, Beer Sheva, Israel; 4 French Associates Institute for Agriculture and Biotechnology, Blaustein Institutes for Desert Research, Ben-Gurion University of the Negev, Sede Boqer Campus, Midreshet Ben-Gurion, Israel

**Keywords:** Desert primary production, Photosynthetic CO_2_ fixation, Growth potential, Evolution, Adaptation, Biogeochemistry, Microscopy, Microbiology techniques, Bacterial physiology, Microbial ecology, Carbon cycle, Carbon cycle

## Abstract

In deserts that cover one-third of the Earth’s terrain, soil cyanobacteria and algae are key carbon dioxide (CO_2_) fixers because few other plants can tolerate intense solar radiation, prolonged desiccation, and extreme temperature shifts. Extensively studied resilience of cyanobacteria and algae to environmental stresses explains their survival in deserts rather than their ubiquitous coexistence. To coexist, prokaryotic cyanobacteria and eukaryotic algae need to grow at similar rates on rare occasions when vital water becomes available. To test that, we incubated illuminated soil samples collected in the Negev desert after rainfall and dewfall events with^14^CO_2_ and microdissected cyanobacteria and algae separately from the soil to determine group-specific CO_2_ fixation potentials. We found that the mean biovolume-specific C-fixation rates of cyanobacteria were 2.5-3.0 times higher than the mean rates of algae, irrespective of the region. These reproducible results suggested that cyanobacteria could grow faster than algae and outcompete them. However, because the carbon content of the cyanobacterial biovolume is ~ 2.5 times higher than the carbon content of the algal biovolume, their mean doubling times are, in fact, similar. Therefore, once it rains, desert cyanobacteria and algae can grow at similar rates, and that explains their recurring coexistence in desert soils.

**Supplementary Information:**

The online version contains supplementary material available at 10.1038/s41598-025-30489-1.

## Introduction

 Prokaryotic cyanobacteria and eukaryotic algae thrive in desert soils^1–4^. When moist and illuminated, these free-living photosynthetic microorganisms use oxygenic photosynthesis, assimilating carbon dioxide (CO_2_) and inorganic salts into organic matter. In deserts, where harsh environmental conditions preclude growth of most vascular plants, cyanobacteria and algae serve as the primary producers of the ecosystem^5^. In fact, primary production across sun-lit non-vegetated terrestrial realms has been driven by the photosynthetic microorganisms since the Neoarchean era. Despite their shared ecological role, prokaryotic cyanobacteria and eukaryotic algae are fundamentally different organisms that belong to two distinct domains of life: Bacteria and Eukarya. Nevertheless, their remarkable capacity to withstand extreme environmental stress and to proliferate during brief periods of favourable conditions enables their cohabitation in desert soils.

Cyanobacteria had evolved oxygenic photosynthesis during the Mesoarchaean (3.2–2.8 billion years ago) and became dominant in virtually all sunlit environments by 2.7 billion years ago^6–8^. Geochemical and isotopic evidence from fossilized terrestrial surfaces indicates that biocrust-like cyanobacterial mats were present as early as ~ 2.6 billion years ago^9,10^. These mats were responsible for terrestrial CO_2_ fixation for nearly a billion years, until the emergence of eukaryotic algae and their subsequent adaptation to subaerial environments^11,12^. The considerably longer evolutionary history of cyanobacteria compared with algae likely conferred them with greater resilience to extreme desert conditions^13^, e.g., intense solar radiation, extreme shifts in temperature and salinity as well as prolonged desiccation^2,14–17^. On the other hand, because algae possess specialized bioenergetic organelles, i.e., mitochondria and chloroplasts, their bioenergetic potential is superior to the bioenergetic potential of prokaryotic cyanobacteria^79,80^. In desert environments, the higher bioenergetic potential of algae may support more rapid growth during brief periods of favourable conditions.

Physiological responses that enable desert cyanobacteria and algae to survive unfavourable environmental conditions were extensively studied in laboratory on cultured isolates—strains of cyanobacteria or algae purified to axenic or unialgal cultures. Both cyanobacterial and algal isolates tolerated extremely high light intensities^18–20^ and exposure to UV radiation^21^. They also survived prolonged heating^22^ and endured desiccation^23–27^. The revealed mechanisms of resistance and defence included accumulation of pigments and photoprotective proteins^20,28^, activation of an antioxidant system^20,27,29^, efficient DNA repair^30,31^, protein recycling^19,32^ as well as down-regulation of damage-prone or potentially damaging cellular processes^20,26,29^, synthesis of intracellular osmolytes^26,30^ and excretion of extracellular polysaccharides^24^. Thus, although cyanobacteria and algae belong to the two different domains of life, their physiological responses to the imposed desert stresses, generally, converge.

Because growth of desert cyanobacteria and algae is governed by moisture availability, it is limited to brief periods during and immediately following sporadic rainfalls, as well as to early-morning hours, after night-time dewfalls, before the dew evaporates^33–35^. Indeed, the maximal photosynthetic activity of biocrust algal and cyanobacterial isolates^36,37^ as well as of cyanobacteria-dominated biocrusts was recorded at low-intermediate temperatures and light intensities^38–41^ typical for cloudy winter days and early morning hours. During the rest of the time, inactive cyanobacteria and algae endure the harsh environmental conditions of the desert until next rain or dewfall.According to the coexistence theory, organisms can coexist when the fitness differences between them are smaller than their niche differences^42^. Because the coexisting cyanobacteria and algae populate same niches within soil biocrusts, their fitness should be similar. The fitness of cyanobacteria and algae at harsh environmental conditions of the desert stems from their ability to synchronize photosynthetic activity and growth with moisture availability^2,43–45^. Whilst stress resistance explained the survival of desert cyanobacteria and algae, their living in deserts should depend on their photosynthetic activity and growth. However, contrary to a relatively coherent set of physiological stress responses, the reported growth rates of cultured desert cyanobacterial and algal isolates ranges widely. Some isolates of cyanobacteria and algae maintain relatively slow growth with doubling times of days to weeks even under optimized culture conditions^38,46–48^. Other isolates achieve doubling times of single hours^49–52^. That is probably not surprising considering the high taxonomic diversity of cyanobacteria and algae in soils^53,54^ and their varied propensities to grow in laboratory conditions. As Allison and colleagues noted back in 1937^36^, “isolates could grow somewhat differently in artificial culture solutions than in nature”. Hence, taxonomic and physiological complexity of soil photosynthetic communities precludes direct extrapolation of growth rates of isolates measured in laboratory conditions to growth *in situ*.

Despite the comparable capacity of cyanobacteria and algae to tolerate environmental stresses, cyanobacteria and algae would not coexist in desert soils unless their average photosynthetic growth rates were similar. A comparison of photosynthetic activity and growth of desert cyanobacteria and algae *in situ* would test that assumption, however, it is challenging technically. The bulk photosynthetic activity could be indirectly assessed by CO_2_ and O_2_ dynamics in and just above soil or approximated by pigment fluorescence of the soil photosynthetic microbes^41,55–57^. Furthermore, photosynthetic CO_2_ fixation rates have been estimated using isotopic^14^C or ^13^C tracers^58–60^. These integral measurements do not, however, allow to compare between photosynthetic activities of cyanobacteria and algae that cohabit the same soil.

Here, we compared photosynthetic activities and growth between natural communities of desert cyanobacteria and algae, which were incubated in soil under conditions close to the desert conditions that support optimal growth of the cells. We expected that cyanobacterial and algal communities that cohabited the soil should, on average, grow at the same rate. The direct comparison of their growth rates was made possible by physical separation of ^14^C-traced cyanobacteria and algae from the same soil sample using microscopy-coupled laser microdissection^61^ followed by ultra-sensitive scintillation counting—a novel approach developed in our laboratory for this study. Because desert cyanobacteria and algae have rich taxonomic diversity and patchy distribution in soils^53,54^, it was infeasible to assess growth rates of each individual species. Therefore, we compared the mean growth rates of cyanobacteria and algae as the representatives of the two domains of life. We sampled the hyper-arid and arid areas of the Negev desert defined based on their potential evapotranspiration-to-precipitation ratio (aridity index, AI) of 0.2 − 0.05 and < 0.05, respectively. These areas of the Negev belong to the Old-World desert belt in the Saharo-Arabian region and, generally, represent global hot desert environments most challenging for growth of cyanobacteria and algae^62^. In order to carry out experiments with physiologically active cyanobacteria and algae, soil samples were collected during or after rainfall or dewfall events in winter and in spring. To achieve the adequate representation of soil microphyte communities, we sampled the dominant terrain features and pooled together randomly collected soil subsamples from each location as well as pooled together several randomly micro-dissected cyanobacterial filaments or algal cells.

## Results

### Characterization of the study sites

**Fig. 1:**
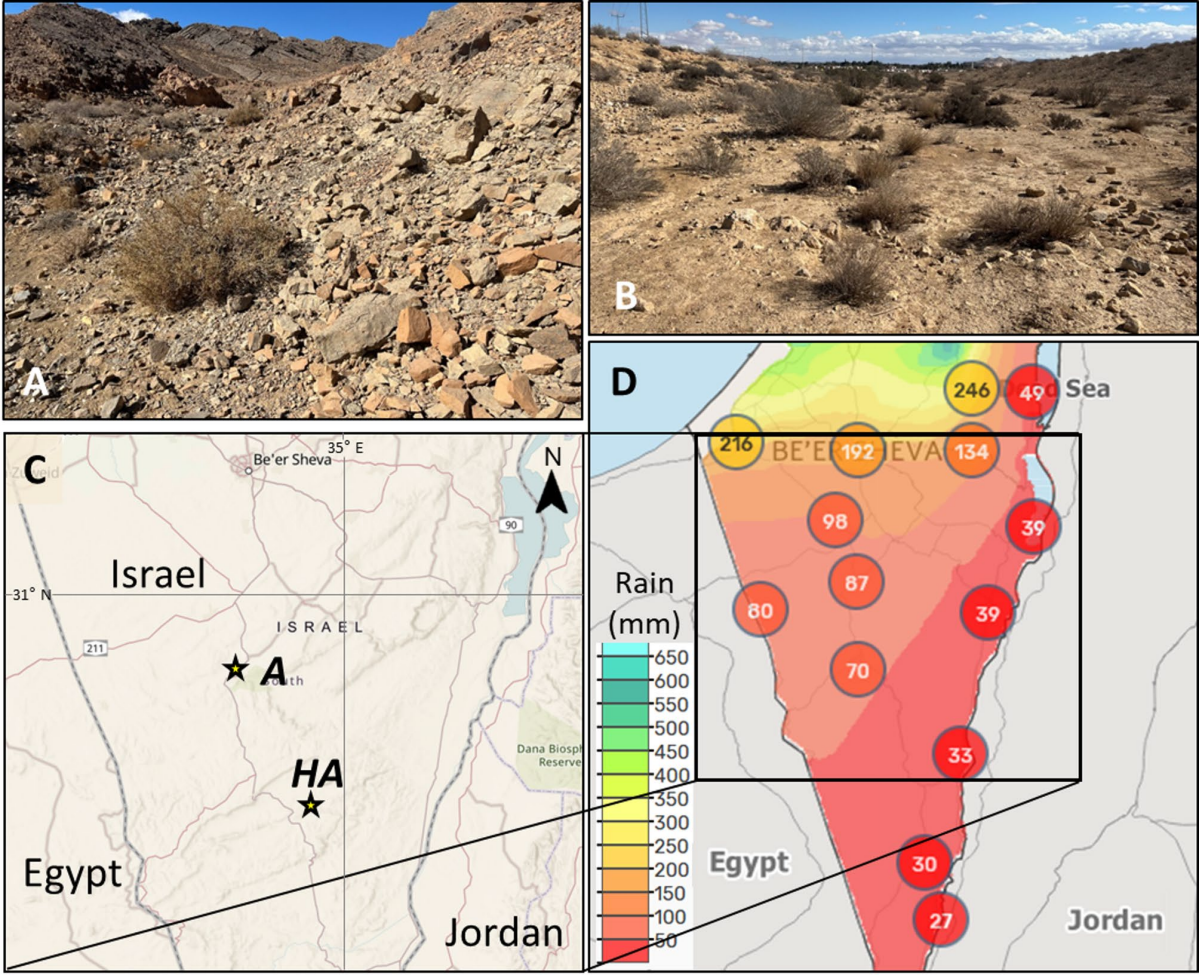
Landscape and location of desert sampling sites. The soil sampling sites in (**A**) the hyper-arid (*HA*) Gevanim Valley, (**B**) the arid (*A*) Halukim Ridge regions of the Negev desert and (**C**) a schematic chart of their geographic locations, based on a cropped topographic map by the U. S. Geological Survey TopoView (https://ngmdb.usgs.gov/topoview). (**D**) A schematic chart of mean annual rainfall (mm) in Israel and the Palestinian Authority from 1981 to 2009 (Israeli Meteorological Service).

In the Negev desert, hot and dry summers and cool winters typically last from April to October and from November to March, respectively. Precipitation generally occurs during winter months and is erratic and varies interannually. The first sampling site (Figs. 1A, C) was in the hyper-arid Gevanim Valley of the Ramon Crater (30° 35’ 46” N, 34° 53’ 24”) at an altitude of ~ 510 m with mean annual precipitation of 40.1 mm (ng.fieldclimate.com) and AI = 0.02^63^. The 10-year (2014–2024, Gevanim Valley Meteorological Station) mean summer and winter temperatures were + 23.9 °C (max + 40.9 °C) and + 14.5 °C (min − 0.4 °C), respectively. The second sampling site (Figs. 1B, C) was on the arid Halukim Ridge (30°51′15′′ N, 34°45′56′′ E), ~ 30 km North to the first site, at an altitude of ~ 480 m with mean annual precipitation of 78.4 mm (ims.gov.il/en/data_gov) and AI = 0.06^64^. The 5-year (2019–2025, Sede Boqer Meteorological Station) mean summer and winter temperatures were + 26.4 °C (max + 42.8 °C) and + 16.6 °C (min + 3.8 °C), respectively.

The surface cover at the two sites (Figs. 1A, B) was bare rocks with pockets of fine-textured desert lithosols and loessial serozems of mostly aeolian origin^65,66^. The organic matter and inorganic carbon contents were significantly higher in soil from the arid site, whereas pH and electrical conductivity of the soils were similar (Supplementary Table 1).

In the arid region, the sparse hillslope vegetation was dominated by *Zybophyllum dumosum*, *Anabasis setifera* and *Astragalus amalecitanus* shrubs, geophytes, e.g. *Urginea maritima* and *Tulipa systola*, and annual grasses, e.g. *Stipa capensis*, whilst *Retama raetam* was common in streambeds. In the hyper-arid region, the growth of the shrubs, geophytes, and grasses was limited to streambeds, which received sufficient runoff from upstream hillslopes and streams whereas the latter lacked vegetation (Figs. 1A, B).

### Comparison of biovolume-specific C-fixation rates between cyanobacteria and algae

Cyanobacterial filaments and algal cells were excised from soil that was collected from the hyper-arid and arid regions in winter and in spring. For the arid region, 28 and 25 samples of cyanobacteria and algae, respectively, were excised in winter and 36 and 37 samples of cyanobacteria and algae, respectively, were excised in spring. For the hyper-arid region, 22 and 18 samples of cyanobacteria and algae, respectively, were excised in spring. Unfortunately, in spring, low abundance of microphytes made their micro-dissection impractical.

We found limited-to-moderate correlation (correlation coefficient *r* = 0.12–0.83) between the cellular biovolumes and ^14^C-fixation rates (Supplementary Fig. 1) for both cyanobacterial filaments and algal cells from either the hyper-arid or arid regions. Namely, their cellular sizes were poor indicators of their photosynthetic growth. This corroborated the wide range of growth rates reported for cultured isolates of desert cyanobacteria and algae^38,46–52^.

**Fig. 2:**
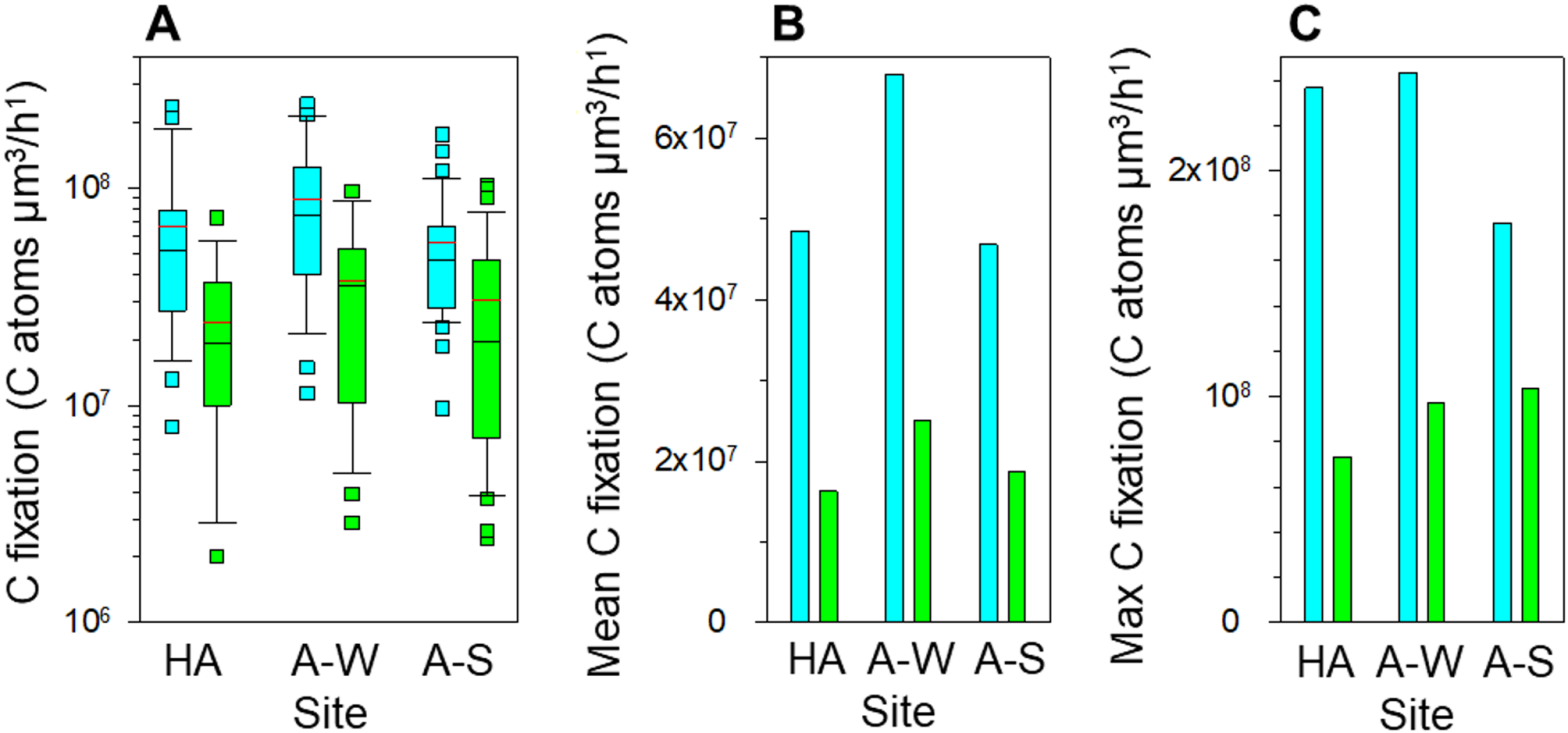
Higher biovolume-specific C-fixation rates of soil cyanobacteria than algae.(**a**) A box plot comparison of biovolume-specific C-fixation rates of soil cyanobacteria (cyan) and algae (green) from the hyper-arid region in winter (HA) and from the arid region in winter (A-W) and spring (A-S). Black and red horizontal lines in boxes indicate the medians and arithmetic means, respectively. To aid comparisons (**b**) the geometric means and (**c**) maxima of the C-fixation rates are shown separately in linear scale.

Next, we aimed to calculate the biovolume-specific C-fixation rates of excised cyanobacteria and algae, i.e., divided the C-fixation rates by corresponding biovolumes (DPM/µm^3^/h). However, to convert the DPM values into more informative values of C atoms fixed by µm^3^ of cellular biovolume in an hour, we had to take in account the ^14^CO_2_ tracer dilution with bioavailable ^12^CO_2_ released from moist soil during the incubation. To assess this dilution and its timing, we studied the release of CO_2_ into the atmosphere by moist desert soil^67^ (See Methods, Supplementary Fig. 2). During the incubation, soil released ~ 2200 ppm of CO_2_ into the chamber atmosphere and diluted ~ 400 ppm of the ^14^C-traced CO_2_ by a factor of 7.2. Assuming that the release of soil CO_2_ and diffusion of atmospheric ^14^C-traced CO_2_ into the soil are approximately linear and reciprocal, soil cyanobacteria and algae would become surrounded by 7.2-fold diluted atmospheric ^14^C-traced CO_2_ only at the end of the incubation. Hence, the incubation average dilution factor would double, 14.4 folds. We used the derived tracer dilution factor and the Avogadro constant to calculate the absolute rates of C-fixation by soil cyanobacteria and algae (Fig. 2, Supplementary Table 2).

The calculated C-fixation rates of cyanobacteria and algae had skewed distributions, i.e., sample variances were larger than the arithmetic mean (Fig. 2A). We normalised these data by applying the logarithmic transformation and compared the geometric means using the unbalanced two-way ANOVA. The geometric means of the biovolume-specific C-fixation rates by cyanobacteria and algae were similar between the regions and seasons (Fig. 2, Supplementary Table 2) irrespective of certain differences in taxonomic composition of cyanobacterial and algal communities (Supplementary Fig. 3). The mean biovolume-specific C-fixation by cyanobacteria was significantly 2.4–2.9 times higher than the mean biovolume-specific C-fixation by algae in both the arid and hyper-arid regions (Fig. 2B). The difference of 1.7–3.2 times between the maximal values of the biovolume-specific C-fixation was even wider (Fig. 2C). Thus, cyanobacteria could fix ~ 2.5 times more C than algae per µm^3^ of their biovolume and, therefore, should outgrow algae in desert soils.

### Biovolume-specific C-content of desert cyanobacteria and algae

The conclusion that cyanobacteria outgrow algae contravened our microscopic observations and results of molecular analyses (Supplementary Figs. 3, 4). In all samples from different regions and seasons cyanobacteria cohabited soils along with algae despite of the apparent capacity of cyanobacteria for faster C fixation and potentially faster growth. To address this controversy, we tested the validity of the assumption that the cellular biovolumes of cyanobacteria and algae served as the equivalent proxies for their biomass. For cyanobacteria, we used published values^68^ of filament biovolume, dry weight, and carbon fraction of a typical filamentous soil cyanobacterium *Leptolyngbya foveolarum*^69^ to calculate a mean biovolume-specific C-content of a filament. For algae, we determined a biovolume-specific C-content of a local algal isolate *Bracteacoccus* sp. We found that the biovolume-specific C-content of a cyanobacterial filament was 2.5 times higher than the C-content of an algal cell, i.e., 7.49 × 10^9^ versus 3.01 × 10^9^ C atoms/µm^3^. This demonstrated that cellular biovolumes of cyanobacteria and algae were poor proxies for their biomass and did not allow us to directly relate the amount of C atoms fixed in a unit of time with the amount of C atoms in cellular biovolume.

### Comparison of potential growth rates of cyanobacteria and algae

**Fig. 3:**
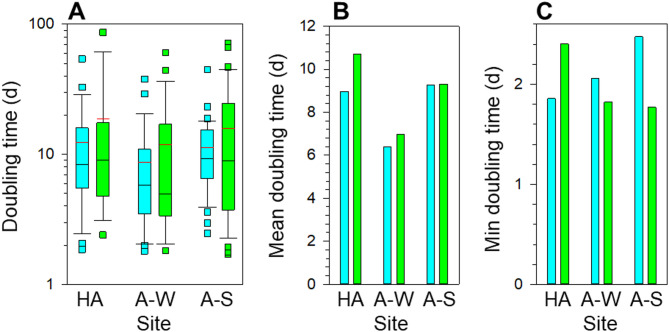
Similar doubling times of soil cyanobacteria and algae. (**a**) A box plot comparison of doubling times of soil cyanobacteria (cyan) and algae (green) from the hyper-arid region in winter (HA) and from the arid region in winter (A-W) and spring (A-S). Black and red horizontal lines in boxes indicate the medians and arithmetic means, respectively. To aid comparisons (**b**) the geometric mean and (**c**) minimal (Min) doubling times are shown separately in linear scale.

Assuming that in soil cyanobacteria and algae photosynthetic CO_2_ fixation was the primary carbon metabolism, we used the biovolume-specific C-content of soil cyanobacteria and algae to compute their potential doubling times (Fig. 3). We then compared geometric means of their doubling times using the ANOVA (Supplementary Table 3). Irrespective of the region, season and differences in taxonomic composition, the mean doubling times of the desert cyanobacteria and algae were statistically similar (Fig. 3B, Supplementary Fig. 3). However, the minimal doubling times differed between cyanobacteria and algae, though the trend changed depending on the region or season (Fig. 3C). Hence, whereas, on average, growth rates of the two groups were similar, it is possible that environmental conditions differentially affected growth of some cyanobacteria and algae.

## Discussion

Desert cyanobacteria and algae are both adapted to tolerate harsh environmental conditions^18–32^ and the living of both is restricted to the periods of moisture availability^33–35^. They both use the energy of sunlight to combine inorganic carbon and salts into organic matter. In calcareous desert soils like those in the Negev, cyanobacteria and algae share microenvironments that, during the periods of moisture availability, provide them with plentiful inorganic carbon (Supplementary Fig. 2) and salts and allow optimal exposure to sunlight^65,70,71^. Hence, the resource availability to soil cyanobacteria and algae is similar. Though grazing of soil mesofauna and gastropods on cyanobacteria and algae in drylands was scarcely studied, consumption of both cyanobacteria and algae was reported^72^. In the light of the governing moisture limitation, we consider that grazing in drylands should be less selective compared with more moist or aquatic environments. Consequently, differences in the group-specific growth potential rates between prokaryotic cyanobacteria and eukaryotic algae could differentiate their fitness. However, because the mean doubling times for the two groups were similar irrespective of the region and season (Figs. 3A, B), fitness of the two groups is, apparently, similar. Therefore, the cyanobacteria and algae which share same desert soil microenvironments coexist because they have similar growth potentials.

The growth potentials were, apparently, independent of taxonomic composition. Though the taxonomic composition of cyanobacterial and algal communities differed between the seasons and regions (Supplementary Fig. 4), their mean doubling times were statistically indistinguishable (Fig. 3B). The determined doubling times are, generally, within the range of the values reported for cultured isolates of desert cyanobacteria and algae^38,46–48^, but lower compared with growth rates of planktonic cells. This difference could be explained by the difference in cell sizes, i.e., because terrestrial cyanobacteria and algae have bigger cell sizes, the estimated doubling times of soil cyanobacteria and algae from the Negev desert are lower than the doubling times of smaller aquatic cyanobacteria and algae^73–75^.

Additionally, comparisons of the cyanobacterial versus algal growth in the extreme terrestrial and aquatic environments diverge. Whilst in the oligotrophic ocean tiny unicellular cyanobacteria can outgrow algae, probably, owing to their high surface area-to-volume ratio^73^, in the desert larger filamentous cyanobacteria and algae grow at apparently similar rates. The capacity of eukaryotic algae to equal growth rates of prokaryotic cyanobacteria in the desert suggests that the superior bioenergetic potential of eukaryotic organisms managed to match the longer evolution of prokaryotic cyanobacteria in the terrestrial realm.

## Conclusions

- The finding that the average doubling time of eukaryotic algae is similar to the doubling time of prokaryotic cyanobacteria despite the longer evolutionary adaptation of the latter to harsh desert environment is an example of convergent physiological evolution of photosynthetic microorganisms that belong to the two domains of life.
- Similarity in the C-normalized C-fixation between cyanobacteria and algae in different desert regions at different seasons indicates that the aridity stress has a limited effect on the photosynthesis *per se* but rather controls the total duration of growth.
- Comparable doubling times of cyanobacteria and algae corroborate their coexistence in soils of the Negev desert and, probably, in other hyper-arid and arid areas similar to those of the Negev desert.

## Methods

### Soil sampling

The hyper-arid Gevanim Valley was sampled on the 26th of January 2022 (11.1 °C, 560 µmol photon/m^2^/s solar radiation,1.6 mm of rainfall on the sampling day). The arid Halukim Ridge was sampled on the 26th of January 2022 (10.0 °C, 480 µmol photon/m^2^/s solar radiation, 2.6 mm of rainfall on the sampling day) and on the 7th of March 2023 (20.4 °C, 1580 µmol photon/m^2^/s solar radiation, three weeks after a 1.1-mm of rainfall, after two nights with dewfall). In total, by the time of sample collection, the hyper-arid region received 32.8 mm of rain (ng.fieldclimate.com), whilst the arid region received 43.0 and 71.6 mm of rain by the two sampling dates, respectively (ims.gov.il/en/data_gov).

To account for spatial heterogeneity, soil samples were collected from two locations on the North-facing slope and from streambeds of three different streams in the hyper-arid region. In the arid region, soil samples were collected from the South-facing and North-facing hill slopes and from a streambed. Samples were collected using a plastic syringe with an orifice of a cylindrical barrel cutoff flat and a piston set to a barrel depth of 2 mm. The syringe was driven into the soil to fill up 2 mm-deep space of the barrel and the excised sample was separated from the underlying substrate by sliding a wide, thin spatula along the barrel opening. The syringe and spatula were accurately lifted together to transfer the sample into a Petri dish. To account for local heterogeneity, samples were collected from ten random points at each location and then mixed. The mixed samples were dried and sieved through a mesh of 2 mm to remove rock fragments and plant material. Then the samples were stored at room temperature until further analyses.

### Measurement of CO_2_ released from soil

To measure the amount of CO_2_ released from moist soil (soil respiration), 12 g of soil were 2-mm sieved to exclude larger mineral particles and plant debris, dispensed into glass vessels, moistened below the field capacity with 3 ml of sterile water and vented for ~ 30 min to let out a CO_2_ emission pulse caused by wetting. After that, glass vessels were sealed with lids equipped with CO_2_ sensors^67^ and incubated in the dark at ambient temperature for ~ 4 days. Accumulation of CO_2_ in the headspace of the vessels was logged every minute (Supplementary Fig. 2).

### C-fixation experiments

To compare rates of C-fixation by cyanobacteria and algae, we incubated the soil samples in air where ambient CO_2_ (~ 400 ppm) was replaced with radiolabelled CO_2_ (^14^CO_2_) at a similar partial pressure^76^. Dry subsamples of 50 mg were dispensed into small, transparent plastic vessels, moistened with sterile, deionised water under non-photosynthetically active green light and kept in the dark overnight to allow soil organism activation. On the following morning, vessels were moistened again to the equivalent of ~ 0.2–0.3 mm precipitation, that supported high rates of CO_2_ fixation^39^ and allowed efficient gas diffusion and placed in a humidified glass bottle together with an open ampule of aqueous, alkaline solution of ^14^C-labelled sodium bicarbonate (NaH^14^CO_3_, specific activity 0.1685 × 10^12^ Bq/mol, pH 10.3, DHI, Denmark). The bottle was sealed with a rubber septum lid and flushed with CO_2_-free air. The solution inside the ampule was then acidified with HPLC-grade concentrated orthophosphoric acid to release gaseous ^14^CO_2_ to the final partial pressure of ~ 400 ppm ^14^CO_2_. The samples in the sealed bottle were incubated at 20–22 °C temperature with 65–70 µmol photon/m^2^/sec of white light because previous studies reported that maximal photosynthetic rates of soil microphytes were achieved in winter, spring and autumn at low-intermediate temperatures and with 15–200 µmol photon m^− 2^ s^− 1^ of illumination^36–41^. Incubations lasted for 45 h to allow sufficient incorporation of the tracer and were terminated by switching off the light. Under non-photosynthetic green light, samples were fixed with paraformaldehyde (2% final concentration) and stored until microscopic analysis and microdissection at + 4 °C in the dark to minimize loss of cellular chlorophyll autofluorescence.

### Micro-dissection of ^14^C-labelled cyanobacteria and algae

For microscopy, the ^14^C-labelled, fixed subsamples in vessels were resuspended in 50 µl of sterile, 0.2 μm filtered, deionised water, and an aliquot of ~ 2 µl of slurry was deposited onto a filter membrane. To spread soil particles, the membrane was rinsed with sterile water, then dried and mounted onto a sample-stage holder of a laser-cutting microscope (LMD7, Leica).

Membranes were examined using long-focus 20×, 40×, and 63× objectives in bright-field and epifluorescence modes. Chlorophyll autofluorescence was visualized using Leica E4 filter system (excitation 436/7 nm, emission > 470 nm). Cyanobacterial and algal cells were identified based on their autofluorescence, presence or absence of chloroplasts and cell morphology. Linear dimensions of each cyanobacterial filament (length and width) or algal cell (diameter) were measured using LMD Application Software calibrated using S1 Micrometer (Electron Microscopy Science, Hatfield, PA). The dimensions were used for calculating cellular biovolumes, assuming that cyanobacterial filaments are cylinders and algal cells are spheres. After imaging, a polygon was drawn around the target filament or cell to guide micro-dissection of the membrane using the focused UV laser beam^61^. The cut-out membrane piece with the filament or cell was gravity-collected in a cap of a 0.2-ml plastic tube with 50 µl of phosphate-buffered saline (PBS, pH 9.0). Success of collection was checked microscopically by examining the cap content using a 5⨯ long-focus objective at bright-field and epifluorescence modes. On average, seven cyanobacterial filaments or eight algal cells were collected in each tube. For negative control, similar-size membrane pieces without cells were micro-dissected and collected in tubes. A total of ~ 50 negative control tubes were prepared and analysed.

**Fig. 4:**
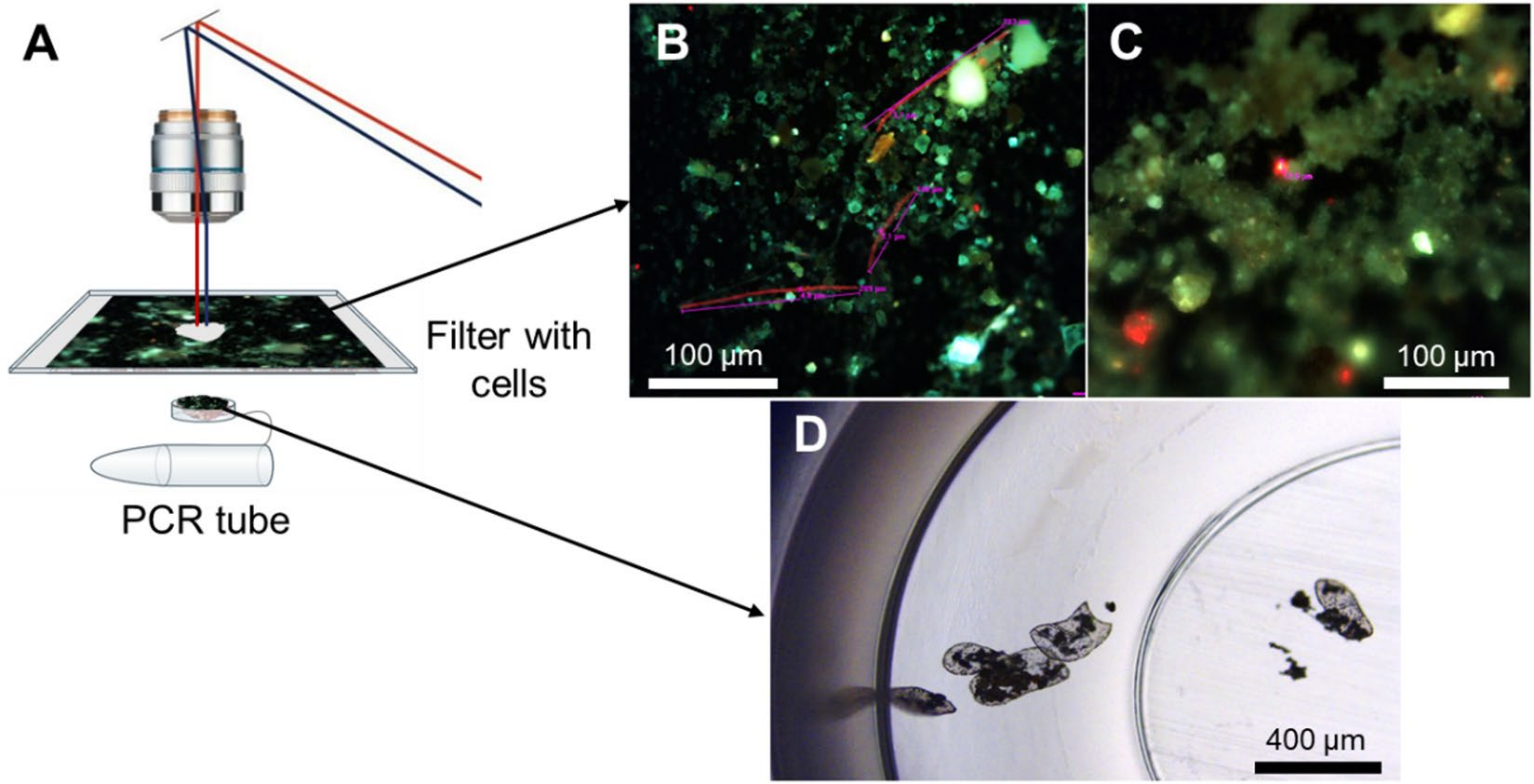
Micro-dissection of ^14^C-labelled cyanobacteria and algae. (**A**) Soil deposited on a filter was examined microscopically, (**B**) cyanobacterial filaments and algal cells were identified using chlorophyll autofluorescence and taxon-specific morphology and their dimensions were measured. (**C**) Filter pieces with the cells of interest were micro-dissected using a focused UV laser and (**D**) gravity-collected into a cap of a PCR tube.

After adding 150 µl of 1 M HCl for acidifying tubes content to release^14^CO_2_, tubes were vented in a fume-hood for 24 h to remove remnants of unfixed inorganic ^14^C. Then each tube was placed into a separate scintillation vial filled with 4 ml of ProSafe scintillation cocktail (Meridian, UK). After equilibration for > 7 days, vials were radio-assayed using a triple-PMT Hidex 300 Super Low scintillation counter (Hidex, Finland). The vial ^14^C-content was quantified as disintegrations per minute (DPM) using a triple-to-double coincidence ratio as an estimate of counting efficiency. The DPM data were matched with the corresponding biovolume data (Supplementary Fig. 1) for subsequent statistical analyses Fig 4.

### Assessment of biovolume-specific carbon content of a representative desert Alga

Conversion of the measured C-fixation rates of soil cyanobacteria or algae into estimates of their doubling times requires knowledge of their mean biovolume-specific carbon content. We calculated this constant for cyanobacteria as 7.49 × 10^9^ C atoms per µm³ based on the carbon content fraction of the dry weight and the dry weight per unit biovolume determined for the model soil cyanobacterium, *Leptolyngbya foveolarum*^68,69^. Because we could not find an analogous constant for model soil algae in literature, we determined it using a local isolate of a green alga, *Bracteacoccus* sp. We chose *Bracteacoccus* sp. because this terrestrial alga is common in deserts worldwide and was repeatedly isolated from the Negev^77,78^, including our laboratory.

Cells of cultured *Bracteacoccus* sp. were harvested from agar plates and suspended in BG-11 media. The mean cellular biovolume and cell concentration were measured by a Multisizer 4e Coulter Counter (Beckman) using a 50-µm aperture. To determine the mean C-content of *Bracteacoccus* cells, their suspension was centrifuged, supernatant aspirated, and the biomass pellet was dried at 70 °C for 48 h. The elemental composition of the dry biomass was measured using an Elemental Analyzer (Thermo FlashSmart CHNS/O). In parallel, aliquots of the cell suspension were filtered and cells collected onto pre-combusted, weighted glass fibre filters (Whatman Grade GF/A, Sytiva). The filters were dried at 70 °C and re-weighted to determine dry weight of the cells collected on the filter. The determined carbon content of 3.01 ± 0.08 × 10^9^ C atoms/µm^3^ of *Bracteacoccus* cells was used to convert the measured C-fixation rates of excised algae into estimates of their doubling times.

### Statistical analyses

Normal distribution of datasets was assessed using the Shapiro-Wilk/Shapiro-Francia tests. Datasets were compared using one-way and two-way analysis of variance (ANOVA). The unbalanced two-way ANOVA was used in most cases because the number of replications differed for each site. Differences were considered statistically significant at *P* < 0.05 and highly significant at *P *< 0.005.

## Supplementary Information

Below is the link to the electronic supplementary material.

Supplementary Material 1

## Data Availability

The datasets generated and/or analysed during the current study are available in the OSF repository: https://doi.org/10.17605/OSF.IO/HQPW7.

## Declarations

### Open research statement

The datasets generated and/or analysed during the current study are available in the OSF repository: 10.17605/OSF.IO/HQPW7.
