# Correction: Similar growth potentials of cyanobacteria and algae explain their coexistence in desert soils

**DOI:** 10.1038/s41598-026-40405-w

**Published:** 2026-02-17

**Authors:** Khin Maw Kyi, Elad Levintal, Nina A Kamennaya

**Affiliations:** 1 Kreitman School of Advanced Graduate Studies, Ben-Gurion University of the Negev, Beer Sheva, Israel; 2 Zuckerberg Institute for Water Research, Blaustein Institutes for Desert Research, Ben-Gurion University of the Negev, Sede Boqer Campus, Midreshet Ben-Gurion, Israel; 3 Goldman Sonnenfeldt School of Sustainability and Climate Change, Ben-Gurion University of the Negev, Beer Sheva, Israel; 4 French Associates Institute for Agriculture and Biotechnology, Blaustein Institutes for Desert Research, Ben-Gurion University of the Negev, Sede Boqer Campus, Midreshet Ben-Gurion, Israel

Correction to: *Scientific Reports* 10.1038/s41598-025-30489-1, published online 01 December 2025

The original version of this Article contained an error in the Introduction section.

“Cyanobacteria had evolved oxygenic photosynthesis during the Mesoarchaean (3.2–2.8 million years ago) and became dominant in virtually all sunlit environments by 2.7 billion years ago.”

now reads:

“Cyanobacteria had evolved oxygenic photosynthesis during the Mesoarchaean (3.2–2.8 billion years ago) and became dominant in virtually all sunlit environments by 2.7 billion years ago.”

The original Article has been corrected.

