# Supplementary material for: Similar growth potentials of cyanobacteria and algae explain their coexistence in desert soils
Source: Sci Rep. 2025 Dec 1;16:830. doi: 10.1038/s41598-025-30489-1 (PMC12780035; doi:10.1038/s41598-025-30489-1)
Supplement: Supplementary file 1 — Supplementary Material 1 [file 41598_2025_30489_MOESM1_ESM.docx]

**Similar growth potentials of cyanobacteria and algae explain their coexistence in desert soils**

Khin Maw Kyi, Elad Levintal, Nina Kamennaya

**Supplementary Fig. 1:** **Comparison between biovolumes (µm^3^) and C-fixation rates (disintegrations per minute, DPM) of cyanobacteria and algae from soil of the Negev desert.**


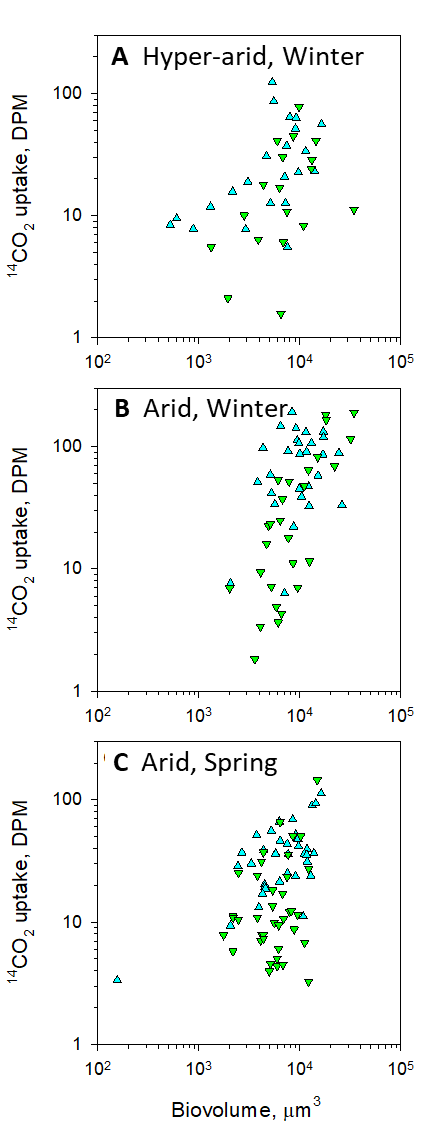


Cyanobacterial filaments and algal cells were excised from soil that was collected from **A** the hyper-arid region in winter and from the arid region **B** in winter and **C** in spring. From the hyper-arid region, there were 22 and 18 samples of excised cyanobacteria and algae, respectively. From the arid region in winter, there were 28 and 25 samples of excised cyanobacteria and algae, respectively. From the arid region in spring, there were 36 and 37 samples of excised cyanobacteria and algae, respectively.

**Supplementary Fig. 2: The reproducible dynamics of CO_2_ release from moist soil into the atmosphere in the replicated experiments S1 and S2.**


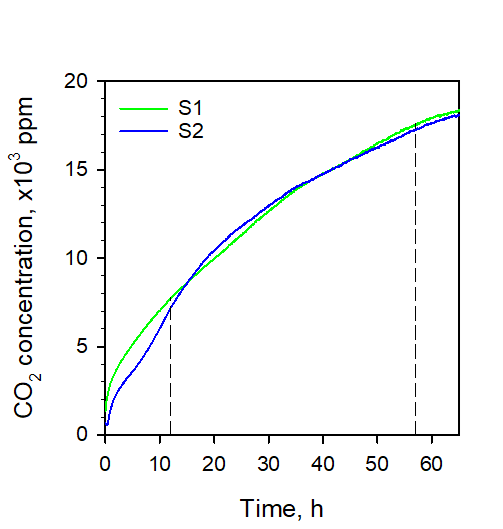


The vertical dashed lines indicate a period chosen for the ^14^CO_2_ fixation experiments.

After an initial, dramatic CO_2_ spike, that followed soil wetting, the soil released CO_2_ at a relatively constant rate for the first ~25 h, then the release rate gradually decreased and leveled off by ~65 h Guided by the dynamics, the incubation time was selected for the 12-57 h period.

**Physico-chemical characterization of soil**

***Methods***

The organic matter content in soil samples was determined using a rapid dichromate oxidation technique [1]. Briefly, sieved and ground soil samples were oxidized with a K_2_Cr_2_O_7_ solution in the presence of concentrated H_2_SO_4_; the remaining Cr_2_O_7_^2-^ was quantified by titration with Fe(SO_4_)(NH_4_)_2_(SO_4_)×6H_2_O terminated on the ferroin indicator color change. The organic matter content was calculated assuming a 76% recovery of organic carbon [2].

Organic materials were heat-extracted from soil using aqueous sodium citrate solution (20 mM, pH 7.0) at 121 °C and 2 bar for 30 min [3]. Following the removal of soil particles, protein and polysaccharide contents of the soil were determined spectrophotometrically using a microplate reader (Infinite 200 PRO, Tecan). Proteins were quantified using the Coomassie method [4]. Polysaccharides were quantified using the phenol-sulfuric acid method [5].

The inorganic carbon content in soil samples was determined using pressure calcimeter method [6]. Briefly, carbonates in sieved and ground soil samples were dissolved in phosphoric acid. The amount of released CO_2_ measured as pressure increase was determined with 2-mbar precision using a pressure transducer (PS100-2bar, MRC) and a digital pressure meter (PS-9302, MRC). The soil content of inorganic carbon was derived from the increase in pressure, which is linearly related to the quantity of dissolved carbonate, using a calibration curve.

***Results***

**Supplementary Table 1**.

Physico-chemical characterization of soil collected from hyper-arid and arid sampling sites. Values are means ± standard error of 5 biological replicates for the hyper-arid site and 3 biological replicates for the arid site, with three technical replicates each. Soil organic matter (SOM), protein, polysaccharide and soil inorganic carbon (SIC) contents differed significantly (two-way ANOVA, *P* < 0.05) between the two locations, whereas pH and electrical conductivity (EC) of the soils were similar between the two locations.

|  | Hyper-arid | Arid | ANOVA *P*-value |
| --- | --- | --- | --- |
| SOC, % | 1.23 ± 0.09 | 1.43 ± 0.007 | 0.006 |
| SIC, % | 34.74 ± 0.66 | 38.54 ± 0.76 | 0.001 |
| Protein, µg/cm^2^ | 5.82 ± 0.834 | 11.12 ± 0.909 | <0.001 |
| Polysaccharide, mg/cm^2^ | 0.492 ± 0.054 | 1.152 ± 0.104 | <0.001 |
| pH | 7.21 ± 0.05 | 6.91 ± 0.23 | 0.1258 |
| EC, µs/cm | 129.46 ± 25.7 | 131.1 ± 27.51 | 0.9675 |

**Statistical analyses**

**Supplementary Table 2**.

Results of unbalanced two-way ANOVA comparison of logarithmically transformed biovolume-specific rates of C-fixation (Log_10_ C atoms/µm^3^/h) by cyanobacteria and algae (Factor A) in the hyper-arid region and in the arid region in winter and in spring (Factor B). <https://www.statskingdom.com/unbalanced-two-way-anova.html>

| Source | Degrees of freedom (DF) | Sum of Square (SS) | Mean Square (MS) | F Statistic (df1, df2) | *P*-value |
| --- | --- | --- | --- | --- | --- |
| Factor A | 1 | 6.2364 | 6.2364 | 45.0572 (1,160) | 3.154E-10 |
| Factor B | 2 | 0.8337 | 0.4168 | 3.0116 (2,160) | 0.05201 |
| Interaction AB | 2 | 0.04661 | 0.02331 | 0.1684 (2,160) | 0.8452 |
| Error | 160 | 22.1455 | 0.1384 |  |  |
| Total | 165 | 29.3117 | 0.1776 |  |  |

***Validation***

*Type*. Most likely, an interaction effect doesn't exist, or only a very small interaction exists.

*Outliers*. Outliers' detection method: Tukey Fence, *k* = 1.5. The data doesn't contain outliers.

*Normality*. The assumption was checked based on the Shapiro-Wilk Test (*a* = 0.05). It is assumed that the residuals do not follow the normal distribution (*P*-value is 0.03311), however, the test is considered robust for moderate violation of the normality assumption. The sample size is greater than 30, hence if the sample data is reasonably symmetric, the statistics may distribute approximately normally.

***Conclusions***

The geometric mean biovolume-specific C-fixation rates by cyanobacteria were significantly higher than the mean fixation rates by algae (Factor A, *P*-values 3.154^-10^) irrespective of the region (Interaction AB, *P*-value 0.8452). The geometric mean biovolume-specific C-fixation rates by cyanobacteria and by algae were similar between the hyper-arid and arid regions (Factor B, *P*-values 0.05201).

**Supplementary Table 3**.

Results of unbalanced two-way ANOVA comparison of logarithmically transformed doubling times (Log_2_ h) by cyanobacteria and algae (Factor A) in the hyper-arid region and in the arid region in winter and in spring (Factor B).

| Source | Degrees of freedom (DF) | Sum of Square (SS) | Mean Square (MS) | F Statistic (df1, df2) | *P*-value |
| --- | --- | --- | --- | --- | --- |
| Factor A | 1 | 0.02446 | 0.02446 | 0.01615 (1,160) | 0.899 |
| Factor B | 2 | 9.1118 | 4.5559 | 3.0076 (2,160) | 0.05221 |
| Interaction AB | 2 | 0.5044 | 0.2522 | 0.1665 (2,160) | 0.8468 |
| Error | 160 | 242.3626 | 1.5148 |  |  |
| Total | 165 | 251.9965 | 1.5273 |  |  |

***Validation***

*Type*. Most likely, an interaction effect doesn't exist, or only a very small interaction exists.

*Outliers*. Outliers' detection method: Tukey Fence, *k* = 1.5. The data doesn't contain outliers.

*Normality*. The assumption was checked based on the Shapiro-Wilk Test (*a* = 0.05). It is assumed that the residuals do not follow the normal distribution (*P*-value is 0.03024), however, the test is considered robust for moderate violation of the normality assumption. The sample size is greater than 30, hence if the sample data is reasonably symmetric, the statistics may distribute approximately normally.

***Conclusions***

The geometric mean doubling times by cyanobacteria and algae were similar (Factor A, *P*-values 0.899) irrespective of the region (Interaction AB, *P*-value 0.8468). The geometric mean doubling times by cyanobacteria and by algae were similar between the hyper-arid and arid regions (Factor B, *P*-values 0.05221).

**Microscopic examination of soil cyanobacteria and algae**

Filamentous cyanobacteria and unicellular algae were the two dominant morphologies found in the examined soil samples. Cyanobacteria were observed either as single filaments or filaments organized into 2D or 3D clusters, with associated mineral grains of varying sizes (Supplementary Fig. 3). Only rarely cyanobacterial filaments contained heterocysts. Algae appeared as single cells or small cell clusters and only rarely at a zoospore stage. No filamentous algae were found.

**Supplementary Fig. 3:** **Light microscopy micrographs of (A-D) algal cells or cell clusters and (E, F) cyanobacterial filaments commonly observed in soil from hyper-arid and arid regions of the Central Negev.**


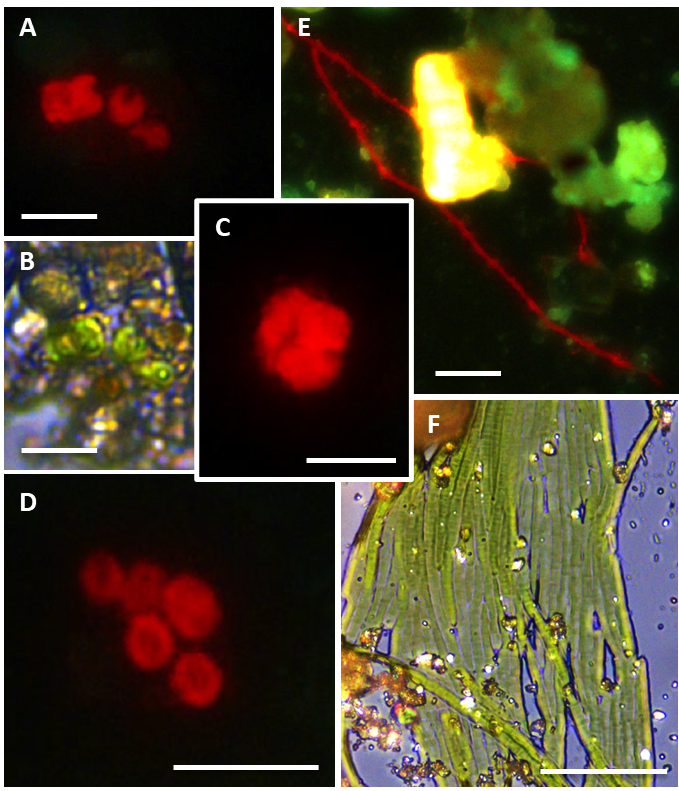


Chlorophyll fluorescence in **A**, **C**-**E** is imaged using a fluorescence mode: excitation at 432-440 nm, emission at >470 nm. Cells in **B** and **F** are imaged using bright-field and DIC modes, respectively. Scale bars are 10 µm for **A**-**D** and 50 µm for **E** and **F**.

**Molecular characterization of cyanobacterial and algal soil communities**

***Methods***

*Soil DNA extraction*

DNA was extracted from 6 g soil following the S protocol for soil DNA extraction [7] modified from Miller et al., 1999 [8]. The extracted DNA was further purified using Quick-Start DNeasy PowerSoil Pro Kit (Qiagen, Germany), and DNA concentration and quality were assessed using the NanoDrop ND-1000 spectrophotometer (ThermoScientific Inc).

*PCR amplification of 16S rRNA gene*

The 1st-stage PCR amplification was carried out using high-fidelity Phanta Flash master mix (Vazyme) and CYA-359F and CYA-781R cyanobacteria-specific primers [9] tailed with CS1 and CS2 adaptors for Amplicon Library preparation [10] (Fluidigm, Access Array Barcode Library for Illumina). Triplicate 10-µl PCR reactions were performed along with the positive and negative controls, which contained either genomic DNA of cultured cyanobacteria or sterile water, respectively. PCR products were visualized using 2 % agarose gel electrophoresis and successful reactions were pooled.

*Library preparation and sequencing*

The pooled PCR products were sent to the Rush Genomics and Microbiome Core Facility (GMCF), where the 2nd-stage PCR was performed in 10 µl reactions using repliQa HiFi ToughMix, with unique dual indices and 1 µl of the 1ststage PCR product was used as a template. PCR products were pooled and cleaned using Pippin Prep with 1.5% agarose gel, targeting DNA fragments (375-750 bp), followed by a 0.6× Ampure cleanup. Sequencing was done at GMCF using an Illumina MiniSeq with a 10% phiX spike-in (2×154 paired-end reads).

*Bioinformatic analysis*

The raw data were deposited to Illumina’s BaseSpace platform as FASTQ files. Primer sequences and adapters were removed from the raw sequences using Cutadapt [11]. Quality filtering, denoising, and removal of chimeric sequences were performed through the DADA2 pipeline, which clustered the sequences into amplicon sequence variants (ASVs) [12]. Taxonomy for the ASVs was assigned using the CyanoSeq (version 1.2) database [13] for cyanobacteria and Greengenes2 [14] and NCBI [15] for algae. Reads identified as Archaea or mitochondria were filtered out.

***Results***

The cyanobacterial community in soil from the hyper-arid and arid regions of the Negev desert was dominated by Leptolyngbyales, Oscilatoriales and Coleofasciculales and comprised less abundant Chroococcidiosidales, Gomontiellales and Chroococcales (Supplementary Fig. 4A, B) [16]. High variability in cyanobacterial community composition between soil subsamples from the hyper-arid region (Supplementary Fig. 4A) reflected higher patchiness. The difference in the community composition between winter and spring in the arid region was relatively minor (Supplementary Fig. 4B). The community composition of algae in the arid region was more variable between the seasons (Supplementary Fig. 4C). Algae from the order of Bacillariales were strongly dominant in winter and the abundance of Prasiolales and, particularly, Chlorosarcinales increased in spring. Because the taxonomic composition of cyanobacterial and algal communities in the Negev is typical for desert soils [17,18], our results could be inferred to hot desert environments in general [16].

**Supplementary Fig. 4: Taxonomic diversity of cyanobacteria and algae in the Negev desert.**


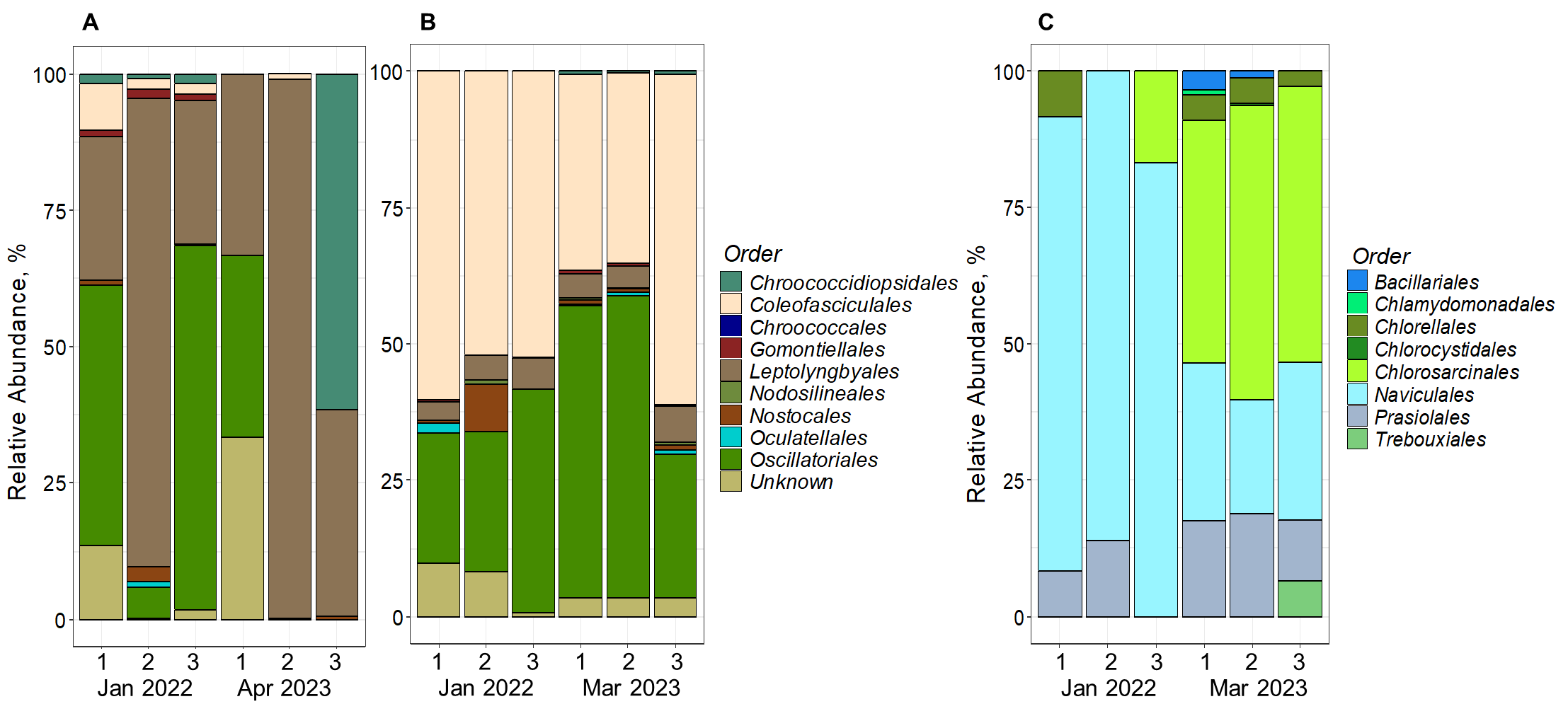


The relative abundance of cyanobacterial 16S rRNA gene sequences in soil collected in winter (Jan 2022) and spring (Apr 2023) from **A** the hyper-arid region and **B** the arid region. **C** The relative algal abundance of chloroplast 16S rRNA gene sequences in soil collected from the arid region in winter and spring.

***References***

1. Nelson, D.W., & Sommers, L.E. Total carbon, organic carbon, and organic matter. In Sparks, D. L., Page, A. L., Helmke, P. A., Loeppert, R. H., Soltanpour, P. N., Tabatabai, M. A., Johnston, C. T. & Sumner, M. E. (eds) *Methods of Soil Analysis*, Part 3, Chemical Methods, Chapter 34, 961–1010 (1996).

2. Walkley, A., & Black, I. A. An examination of the Degtjareff method for determining soil organic matter and a proposed modification of the chromic acid titration method. *Soil Sci.* **37**, 29–38 (1934).

3. Wright, S. F., & Upadhyaya, A. Extraction of an abundant and unusal protein from soil and comparison with hyphal protein of arbuscula mycorrhizal fungi. *Soil Sci*, **161**, 575–586 (1996).

4. Bradford, M. M. A rapid and sensitive method for the quantitation of microgram quantities of protein utilizing the principle of protein-dye binding. *Anal. Biochem*, **72**, 248–254 (1976).

5. Zavřel, T., Očenášová, P., Sinetova, M., & Červený, J.. Determination of storage (starch/glycogen) and total saccharides content in algae and cyanobacteria by a phenol-sulfuric acid method. *Bio‑Protoc*, **8**, 1–13 (2018).

6. Loeppert, R. H., & Suarez, D. L. Carbonate and gypsum. In Sparks, D. L., Page, A. L., Helmke, P. A., Loeppert, R. H., Soltanpour, P. N., Tabatabai, M. A., Johnston, C. T. & Sumner, M. E. (eds) Methods of Soil Analysis, Part 3, Chemical Methods, Chapter 15, 437–474 (1996).

7. Sagova-Mareckova, M., Cermak, L., Novotna, J., Plhackova, K., Forstova, J., & Kopecky, J. Innovative methods for soil DNA purification tested in soils with widely differing characteristics. *Appl. Environ. Microbiol.* **74**, 2902–2907 (2008).

8. Miller, D. N., Bryant, J. E., Madsen, E. L. & Ghiorse, W. C. Evaluation and optimization of DNA extraction and purification procedures for soil and sediment samples. *Appl. Environ. Microbiol.* **65**, 4715–4724 (1999).

9. Nübel, U., Garcia-Pichel, F., & Muyzer, G. PCR primers to amplify 16S rRNA genes from cyanobacteria. *Appl. Environ. Microbiol.* **63**, 3327–3332 (1997).

10. Naqib, A., Poggi, S., Wang, W., Hyde, M., Kunstman, K., & Green, S. J. Making and sequencing heavily multiplexed, high-throughput 16S ribosomal RNA gene amplicon libraries using a flexible, two-stage PCR protocol. *Gene Expr. Anal. Methods Protoc.* **1783**, 149–169 (2018).

11. Martin, M. Cutadapt removes adapter sequences from high-throughput sequencing reads. *EMBnet J.* **17**, 10–12 (2011).

12. Callahan, B. J., McMurdie, P. J., Rosen, M. J., Han, A. W., Johnson, A. J. A. & Holmes, S. P. DADA2: High-resolution sample inference from Illumina amplicon data. *Nat. Methods* **13**, 581–583 (2016).

13. Lefler, F. W., Berthold, D. E., & Laughinghouse IV, H. D. Cyanoseq: A database of cyanobacterial 16S rRNA gene sequences with curated taxonomy. *J. Phycol.* **59**, 470–480 (2023).

14. McDonald, D. et al. Greengenes2 unifies microbial data in a single reference tree. *Nat. Biotechnol.* **42**, 715–718 (2024).

15. Federhen, S. The NCBI taxonomy database. *Nucleic Acids Res.* **40**, D136–D143 (2012).

16. Kyi, K. M., Zubkov, M. V. & Kamennaya N. A. A minority of desert cyanobacteria and algae is responsible for the bulk of CO_2_ fixation. *Physiol. Plant.* 177, e70634 (2025).

17. Treves, H. *et al.* Metabolic flexibility underpins growth capabilities of the fastest growing alga. *Curr. Biol.* **27**, 2559–2567 (2017).

18. Dvořák, P. *et al.* *Modern Topics in the Phototrophic Prokaryotes: Environmental and Applied Aspects*. (Springer, 2017).
